# A Narrative Overview of Fatal Myocarditis in Infant with Focus on Sudden Unexpected Death and Forensic Implications

**DOI:** 10.3390/jcm14124340

**Published:** 2025-06-18

**Authors:** Matteo Antonio Sacco, Saverio Gualtieri, Maria Cristina Verrina, Valerio Riccardo Aquila, Lucia Tarda, Alessandro Pasquale Tarallo, Isabella Aquila

**Affiliations:** Department of Medical and Surgical Sciences, Institute of Legal Medicine, University “Magna Graecia” of Catanzaro, Viale Europa, Loc. Germaneto, 88100 Catanzaro, Italy; matteoantoniosacco@gmail.com (M.A.S.); saverio.gualtieri@studenti.unicz.it (S.G.); mariacristina.verrina@studenti.unicz.it (M.C.V.); valerioriccardo.aquila@studenti.unicz.it (V.R.A.); lucia.tarda@studenti.unicz.it (L.T.); alessandropasquale.tarallo@studenti.unicz.it (A.P.T.)

**Keywords:** myocarditis, sudden infant death syndrome (SIDS), pediatric sudden death, forensic pathology, autopsy findings, lymphocytic myocarditis

## Abstract

Myocarditis, an inflammatory disease of the myocardium, is increasingly recognized as a potential contributor to sudden infant death syndrome (SIDS), though often underdiagnosed. This study reviews the current literature on the association between myocarditis and sudden death in infants, with a focus on autopsy and histopathological findings. A comprehensive search of the PubMed database yielded 64 studies published between 1960 and 2024; after applying specific inclusion criteria—such as patient age (0–6 years), presence of autopsy data, and forensic investigation—40 studies were analyzed in detail. The review identified myocarditis—especially lymphocytic—as an underrecognized but critical cause of sudden death in infants and children. Histological, molecular, and immunohistochemical findings highlighted viral infections, immune dysregulation, and structural anomalies as frequent etiological factors. Several SIDS cases were reclassified as myocarditis upon in-depth examination. These findings underscore the value of standardized autopsy protocols and integrated diagnostic approaches. Advanced postmortem diagnostic techniques, including polymerase chain reaction (PCR) and immunohistochemistry, have enhanced the detection of viral myocarditis. In addition, structural cardiac anomalies, such as cardiomyopathies and coronary abnormalities, may co-exist and contribute to sudden cardiac death. These findings emphasize the need for standardized autopsy protocols and the integration of molecular diagnostics in forensic investigations of SIDS. Further research is essential to improve early detection, refine diagnostic criteria, and develop preventive strategies to reduce the incidence of sudden infant death related to myocarditis.

## 1. Introduction

### 1.1. Introduction to Myocarditis

Myocarditis, an inflammatory disease of the myocardium, poses significant clinical challenges due to its diverse etiology, variable presentation, and potential for serious complications. This condition can arise from a multitude of causes, including infectious agents, autoimmune responses, and toxic exposures, necessitating a nuanced classification system based on both etiology and histological patterns [1,2]. Understanding these classifications is crucial, as they directly influence treatment strategies and prognostic outcomes. Epidemiologically, myocarditis exhibits varying prevalence across different populations, influenced by demographic factors such as age, gender, and geographical location, which underscores the need for tailored public health approaches. Clinically, myocarditis manifests with a spectrum of symptoms ranging from mild to life-threatening, and its presentation can differ significantly among age groups, particularly in infants who may exhibit unique clinical characteristics and complications [3,4]. Accurate diagnosis remains a critical hurdle in managing myocarditis, given the overlapping signs with other cardiac conditions and the limitations of standard diagnostic tests. Advanced imaging modalities play a pivotal role in enhancing diagnostic accuracy, yet challenges persist in differentiating myocarditis from other myocardial disorders. Treatment options have evolved, incorporating conventional pharmacological strategies and, in some cases, immunomodulatory approaches; however, the management of infants requires specialized protocols to address their distinct physiological needs and vulnerability to rapid deterioration. Through a comprehensive exploration of these facets—classifications, epidemiology, clinical characteristics, diagnostic challenges, and treatment options—this paper aims to provide a thorough understanding of myocarditis, highlighting its complexity and the imperative for ongoing research to improve outcomes for affected individuals, particularly the vulnerable infant population.

### 1.2. Classifications of Myocarditis

Myocarditis is a complex condition, with multiple etiological categories that reflect its diverse pathophysiological origins. Primarily, classic myocarditis arises from the host’s immune response against infectious organisms, which include not only common viral agents but also less frequent bacterial, protozoal, or fungal pathogens [1,2]. These infectious agents initiate an inflammatory process in the myocardium, which can persist even after the clearance of the initial infectious organisms, leading to chronic inflammatory events within the heart tissue [1]. Beyond infectious causes, myocarditis can develop as a hypersensitivity reaction, where the immune system overreacts to certain drugs or environmental agents, resulting in inflammation of the myocardium [1]. Additionally, systemic diseases characterized by immune dysregulation are known to cause myocarditis, as the inflammation may either be triggered or exacerbated by autoimmune processes [1]. This is particularly evident in conditions where the immune response is misdirected against cardiac tissues, leading to inflammatory cardiomyopathy. Furthermore, exposure to various toxic substances and drugs can precipitate myocarditis, either as a direct toxic reaction or through modulation of the immune response [1,2]. Understanding these diverse etiological factors is crucial for accurate diagnosis and effective management of myocarditis, emphasizing the need for targeted interventions that address the specific underlying cause in each patient.

The classification of myocarditis according to histological patterns is primarily centered around the presence and type of inflammatory infiltrates within the myocardial tissue. One of the fundamental classifications involves identifying a dense infiltrate of lymphocytes, which is a hallmark of lymphocytic myocarditis [3]. These criteria emphasize the need for histological examination to confirm the presence of lymphocytes in direct association with myocyte damage and necrosis, a critical finding in diagnosing and distinguishing myocarditis from other cardiomyopathies [3]. This histological approach not only aids in diagnosis but also guides treatment strategies, highlighting the importance of accurate classification in managing myocarditis effectively. As the understanding of histological patterns continues to evolve, there is a pressing need for ongoing research and refinement of classification criteria to improve patient outcomes and tailor therapeutic interventions more precisely. This need becomes particularly relevant in the pediatric and infant population, where the absence of necrosis does not exclude lethality, particularly when the cardiac conduction system is involved.

### 1.3. Epidemiology of Myocarditis

The prevalence of myocarditis varies significantly across different populations, influenced by factors such as age, sex, ethnicity, and external triggers like infections and vaccinations. Classical viral-infection-related myocarditis is more frequently observed in males from childhood through young adulthood [4,5,6]. This gender disparity may be linked to hormonal and immunological differences that influence susceptibility to myocarditis following infections and vaccinations [6]. Conversely, women are more affected by myocarditis in the postmenopausal phase, indicating that hormonal changes over a lifespan significantly impact myocarditis prevalence [6]. Ethnic differences also play a crucial role; African Americans, for instance, experience a higher morbidity and mortality rate from COVID-19, which could correlate with an increased incidence of myocarditis in this group [7]. Additionally, myocarditis has been observed following vaccinations, particularly with the COVID-19 mRNA vaccines like Comirnaty and Spikevax, predominantly affecting younger males; however, these cases are generally mild and self-limiting [6]. Understanding these demographic and biological factors is essential for tailoring public health interventions and treatment strategies to mitigate the risk and manage cases of myocarditis effectively across diverse populations.

Demographic factors significantly influence the incidence of myocarditis, with gender and age being primary determinants. Notably, males have consistently shown higher age-standardized rates (ASRs) of myocarditis compared to females from 1990 to 2019, underscoring the gender disparity in disease incidence [8]. Additionally, age plays a critical role, as the burden of myocarditis varies across different age groups, with notable peaks observed in specific demographics [8]. In 2019, high incidents of cases and deaths were recorded among senior citizens of both sexes, highlighting the increased vulnerability of this age group to myocarditis [8]. Furthermore, the age-standardized incidence rates (ASIRs) were more pronounced in high sociodemographic index (SDI) regions, suggesting that socioeconomic factors also contribute to the patterns of myocarditis incidence [8].

Geographical variations significantly impact the epidemiology of myocarditis, as evidenced by the differential prevalence and types of viral pathogens detected across various regions. In North America and Europe, viral infections are predominantly identified as the primary cause of acute myocarditis, with Parvovirus B19 (PVB19) and human herpesvirus 6 (HHV6) emerging as the most frequently detected viruses in recent years [9]. This shift in viral pathogens has been observed over the past two decades, suggesting a temporal and geographical evolution in the virological landscape of myocarditis [9]. Conversely, in Africa, while viral myocarditis remains the most common form, the specific pathogens and their epidemiological trends are less well-defined, with studies indicating that enteroviruses and adenoviruses, once common culprits, are now notably absent in certain cohorts [9]. The parallels in pathogen range between Africa and developed regions, however, underscore a complex interplay between geographic factors and viral prevalence [9]. Therefore, further research is essential to elucidate the intricate mechanisms driving these geographical variations, which could inform targeted public health strategies and improve diagnostic and therapeutic approaches for myocarditis globally.

### 1.4. Clinical Characteristics of Myocarditis

The clinical presentation of myocarditis is notably diverse, reflecting its complex pathophysiology and varying impact on cardiac function. Patients may experience a broad spectrum of symptoms, ranging from completely asymptomatic states to severe cardiac complications such as sudden cardiac death due to cardiogenic shock and malignant arrhythmias [10]. One of the key challenges in diagnosing myocarditis lies in its polymorphic symptomatology, where signs can vary significantly based on the stage of the disease and the individual’s response to the inflammatory process [10]. For instance, while some individuals might report a history of flu-like symptoms preceding the onset of myocarditis, others might directly present with acute heart failure or arrhythmias without any prior warning signs [4]. This variability underscores the need for clinicians to maintain a high index of suspicion and employ comprehensive diagnostic evaluations when confronted with recent onset cardiac symptoms, especially in young patients and infants.

Fulminant myocarditis represents a severe and life-threatening form of myocarditis, often leading to significant complications such as multiorgan failure and cardiogenic shock. The rapid progression of inflammation and cardiac dysfunction in fulminant myocarditis can result in compromised heart function, which in turn may precipitate a cascade of systemic failures impacting various organ systems [11,12]. As the heart struggles to maintain adequate blood circulation, vital organs may suffer from insufficient perfusion, leading to multiorgan failure [12]. The interconnectedness of these complications underscores the critical need for prompt diagnosis and aggressive management to prevent irreversible damage and improve patient outcomes [13,14,15,16,17,18,19,20,21,22,23,24,25,26,27,28,29,30,31,32,33,34,35,36,37,38,39,40,41,42,43,44,45,46,47,48,49]. Timely interventions, such as mechanical circulatory support or heart transplantation, may be necessary to stabilize patients and mitigate the severe consequences associated with this condition [49,50,51,52,53,54,55,56,57,58,59,60,61,62,63,64,65,66,67,68,69,70,71,72,73,74,75,76,77,78,79]. Emerging evidence supports the early use of endomyocardial biopsy (EMB) as a diagnostic tool in acute clinical scenarios [80,81,82,83,84,85,86,87,88,89]. EMB allows the in vivo histological confirmation of myocardial inflammation, guiding targeted immunosuppressive or antiviral therapy when appropriate. Recent studies have demonstrated its safety and diagnostic yield even in critically ill patients, including in the context of mechanical circulatory support [90,91,92,93,94,95,96,97,98,99,100,101,102,103,104,105,106,107,108]. The FOOLMOON study further supports its implementation in fulminant myocarditis for timely etiology-based intervention [109].

### 1.5. Diagnosis of Myocarditis

The diagnostic evaluation of myocarditis involves a comprehensive approach that integrates a variety of tests to accurately identify and assess the condition. Endomyocardial biopsy (EMB) is considered the gold standard for diagnosing myocarditis, particularly in cases where the diagnosis remains unconfirmed through other methods [10]. Despite its invasive nature, EMB provides direct histological evidence and, when combined with PCR, can detect viral genomes within myocardial tissue, thereby enhancing diagnostic accuracy [13]. However, due to its invasiveness and sampling limitations, noninvasive techniques like cardiac magnetic resonance (CMR) have gained prominence. CMR is currently regarded as the noninvasive gold standard for diagnosing myocarditis, offering detailed imaging that reveals myocardial edema and injury through updated Lake Louise criteria [10]. Echocardiography remains a useful adjunct, particularly for ruling out structural heart disease and detecting ventricular dysfunction. These diagnostic modalities offer complementary insights, enhancing the ability to distinguish myocarditis from other causes of cardiac dysfunction.

Accurately diagnosing myocarditis presents several challenges, predominantly due to its heterogeneous clinical presentation and diverse etiological factors [10,14,15]. The protean nature of symptoms often overlaps with other cardiac conditions, necessitating careful differential diagnosis to rule out acute coronary syndrome and other similar presentations [15,16]. Despite advances in imaging techniques such as CMR, interpretation bias and the need for expert analysis may hinder timely diagnosis. These challenges underscore the need for integrated diagnostic approaches and the development of standardized protocols to ensure accurate and early recognition, particularly in pediatric and infant cases where clinical signs may be subtle or absent.

### 1.6. Treatment Options and Infant-Specific Implications

The treatment of myocarditis is multifaceted and often necessitates a personalized approach due to the heterogeneous nature of the disease [17]. Corticosteroids serve as a cornerstone in therapy for some subtypes, such as eosinophilic myocarditis, with steroid-sparing agents used to enhance treatment efficacy and reduce adverse effects [17]. The role of immunosuppressive therapies continues to evolve, with mixed evidence supporting their use, thus highlighting the need for further trials.

In infants, myocarditis frequently presents with a more severe clinical course and poorer prognosis. Treatment protocols must account for developmental differences in immune function and myocardial response. The condition is often associated with progression to dilated cardiomyopathy (DCM), and in severe cases, heart transplantation remains the only viable option [24]. Myocarditis accounts for a significant percentage of pediatric DCM and is a leading indication for cardiac transplantation in children without congenital anomalies [24]. These findings underscore the need for timely diagnosis and early, aggressive intervention in infants to mitigate the risk of irreversible cardiac damage and improve long-term outcomes.

### 1.7. Sudden Infant Death Syndrome

**Figure 1:**
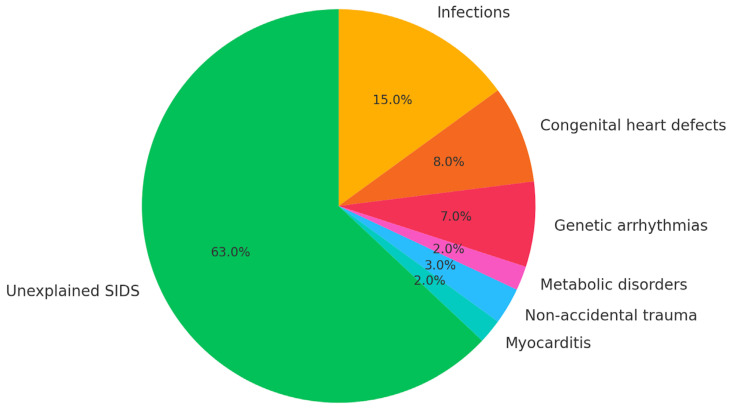
Estimated distribution of identifiable causes in sudden infant death cases, based on data from retrospective autopsy-based studies.

Sudden infant death syndrome (SIDS) is defined as the sudden and unexpected death of an infant under one year of age that remains unexplained after a thorough case investigation, including complete autopsy, examination of the death scene, and review of clinical history. It remains a leading cause of post-neonatal mortality in high-income countries, despite declining trends over recent decades. The etiology of SIDS is considered multifactorial, and current conceptual models—such as the “triple risk model”—propose an interaction between a vulnerable infant, a critical developmental period, and an exogenous stressor (e.g., prone sleeping, overheating, infection). Accurate postmortem evaluation is essential to distinguish SIDS from other identifiable causes of death, including metabolic disorders, cardiac channelopathies, infections, and structural anomalies (Figure 1).

Current international guidelines for the evaluation of sudden unexpected infant death (SUID), including cases classified as SIDS, emphasize the importance of a comprehensive, multidisciplinary protocol. This includes a complete autopsy with histological and toxicological analysis, detailed investigation of the death scene (using doll reenactments if necessary), and collection of the infant’s medical and family history. Recommendations issued by bodies such as the CDC (Centers for Disease Control and Prevention), the American Academy of Pediatrics, and the European Society of Pathology converge in stressing the need for uniform classification and cause-of-death coding [106,107]. The use of postmortem ancillary testing (e.g., microbiology, molecular genetics, metabolic screening) is increasingly advocated in unexplained cases.

## 2. Materials and Methods

A narrative review of the literature in which Pubmed was used as a search engine for articles that addressed the topic of sudden cardiac death in pediatric patients with myocarditis was carried out. Keywords used were “myocarditis and sudden death in infants and autopsy”. It proceeded in two phases: the first phase was the reading of abstracts; the second phase was the reading of full article. Each study was selected after careful reading of the abstract, and all types of work were included, such as case reports, case series, comparative studies, observational studies, reviews, systematic reviews, and meta-analyses that treated sudden cardiac death in pediatric patients aged 0–6 years with myocarditis. Studies investigating SIDS, in particular its incidence and etiology, were included. Additional criteria for inclusion were the presence of autoptic evidence, forensic studies, and clinical studies with diagnostic evidence of death in hospital. After reading the abstracts, studies that did not meet the inclusion criteria mentioned above were discarded, and the entire articles whose abstracts met the inclusion criteria were read. Once the articles had been examined, following the inclusion criteria, it was finally decided to exclude articles that did not meet these criteria. Finally, the studies were compared.

**Figure 2:**
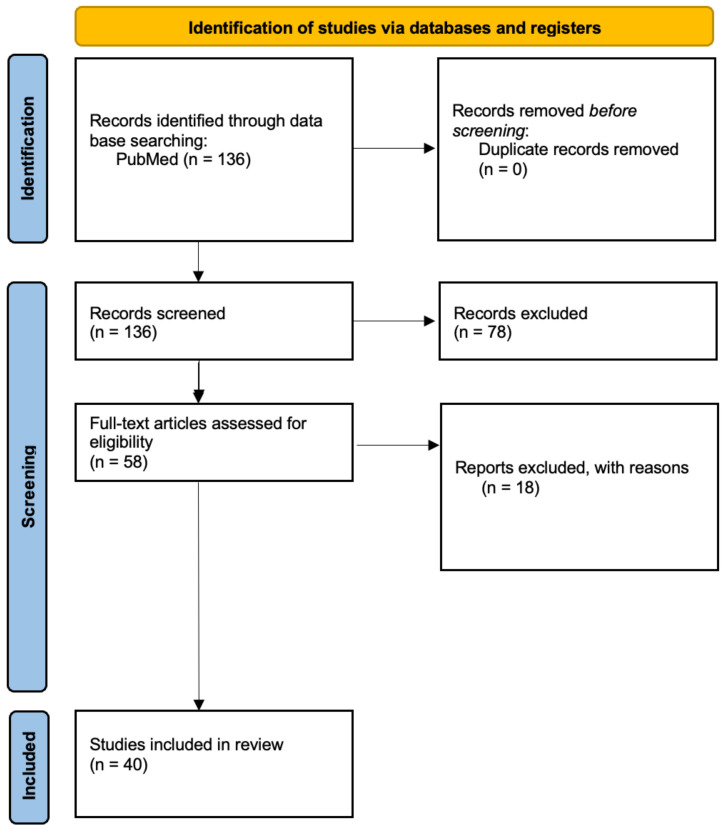
Flow diagram about selection process.

Although no formal assessment of publication bias was performed, given the narrative nature of the review, efforts were made to ensure balance by including both confirmatory and nonconfirmatory findings across different study designs. A simplified flow diagram illustrating the selection process is provided (Figure 2).

The selected studies were subsequently reviewed and qualitatively analyzed in order to identify recurrent pathological patterns, diagnostic findings, and methodological approaches relevant to the role of myocarditis in pediatric sudden death.

## 3. Results

### Review Results

The analysis of the 40 selected articles reveals a complex and multifactorial relationship between myocarditis and sudden death in the pediatric population, particularly among infants under one year of age. Numerous studies emphasize that myocarditis—especially in its lymphocytic form—remains significantly underdiagnosed and may represent a critical cause of sudden and unexpected death in this age group. Dettmeyer et al. support this interpretation through detailed autopsy-based investigations that underscore the frequent presence of lymphocytic myocarditis in pediatric fatalities [47].

Efforts to improve diagnostic accuracy have led to the development of more structured postmortem protocols. For instance, Bakker et al. proposed a comprehensive approach integrating clinical, pathological, and genetic data to elucidate the etiology of sudden cardiac arrest in children, which can also guide preventive measures for surviving relatives [70]. Autopsy series conducted in different geographical areas, such as the studies by Vassalini et al. in Italy and Fragkouli et al. in Greece, report that cardiomyopathies and congenital coronary anomalies are among the most frequent causes of sudden death in children and individuals aged 1–40 years [71,78].

Infectious agents, particularly viruses, also play a major etiological role. Dauger et al. documented a fatal case of myocarditis associated with Varicella Zoster virus [72], while Yagmur et al. and Shimizu et al. used molecular diagnostics to detect viral genomes in myocardial tissue of SIDS victims, supporting a viral origin in at least a subset of cases [80,87]. Okuni et al. further expanded on this by discussing immunopathological mechanisms, including thymic alterations and immune-mediated injury, that may contribute to the chronic evolution of myocarditis in pediatric patients [90].

The utility of immunohistochemistry in enhancing diagnostic sensitivity was demonstrated by Grasmeyer et al., who showed that inflammatory markers such as CD3 and CD68 allow for more accurate detection of myocardial inflammation that may be missed on routine histology [37]. Several studies also underscore the diagnostic overlap between myocarditis and sudden infant death syndrome (SIDS). Cases initially classified as SIDS were subsequently reinterpreted as myocarditis following deeper histopathological or molecular re-examination, as reported by Shatz et al., Dettmeyer et al., and Byard [83,95,102]. This ambiguity reinforces the need for clear classification criteria, as advocated by Byard [81], and for the consistent application of standardized autopsy protocols, such as those proposed by Rizzo et al. [41].

In parallel, structural cardiac anomalies remain an important category of causes. Tavora et al. and Doolan et al. identified congenital coronary anomalies and cardiomyopathies as leading contributors to sudden death in children and adolescents [79,84], a finding further supported by Smith et al. and Kon et al., who emphasized the importance of thorough cardiac evaluation even in patients without prodromal symptoms [74,86]. Other reports broaden the spectrum of differential diagnoses, including incomplete Kawasaki disease [91,99], giant cell myocarditis [93], polyarteritis nodosa [94], and nonspecific forms of myocarditis [89], each of which may mimic more common infectious or cardiac conditions and complicate the diagnostic process.

The importance of molecular autopsy techniques is highlighted in studies such as that by Skinner et al., who identified pathogenic mutations in long QT-related genes in cases of unexplained pediatric death [88]. Large retrospective population studies, including those by Diaz et al., Ilina et al., and Morentin et al., provide broader epidemiological context, showing a consistent presence of undiagnosed myocarditis and other cardiac pathologies in pediatric cohorts [75,77,82].

Comorbidities may also influence the fatal outcome of myocarditis, as seen in the case described by Brady et al., where CMV-induced pancarditis occurred in a neonate with AIDS [103], or in the report by Lajoie et al., where acute myocarditis was associated with Reye’s syndrome and led to sudden cardiovascular collapse [105].

Taken together, the data underscore the critical role of integrating clinical history, histopathological evidence, molecular diagnostics, and standardized postmortem protocols to accurately identify myocarditis as a cause of sudden death in infants and children, to distinguish it from clinically and morphologically similar conditions, and to support both forensic casework and public health initiatives (Table 1 and Table 2).

## 4. Discussion

The systematic review of 40 studies revealed that myocarditis, particularly in its lymphocytic form, is an underdiagnosed but significant contributor to sudden death in infants and young children. Histopathological and molecular investigations, including immunohistochemistry and viral genome detection, consistently identified myocardial inflammation and infectious agents such as enteroviruses and Varicella Zoster virus. Several cases initially classified as SIDS were later reinterpreted as myocarditis upon deeper analysis, underscoring diagnostic ambiguity and the need for standardized autopsy protocols. Structural cardiac anomalies, genetic mutations (e.g., long QT syndrome), and rare inflammatory conditions were also identified as potential causes. Overall, the integration of clinical, pathological, and molecular data is essential for the accurate postmortem diagnosis of myocarditis and the differentiation from other mimicking conditions.

Although the reported prevalence of myocarditis in SIDS cohorts is generally low (1–2%), this figure likely reflects both underdiagnosis and methodological heterogeneity across studies. In many cases, myocarditis was identified only through retrospective review or the use of advanced techniques such as immunohistochemistry and molecular virology. Importantly, even if myocarditis accounts for a limited proportion of SIDS cases, its presence carries significant diagnostic, preventive, and medico-legal implications. The detection of myocardial inflammation provides a tangible, potentially actionable explanation for otherwise unexplained deaths, warranting continued integration of myocarditis assessment in SIDS investigation protocols.

### 4.1. Epidemiology of Sudden Infant Death Syndrome (SIDS)

SIDS remains one of the most perplexing and tragic phenomena in pediatric health, representing a significant public health concern that claims the lives of approximately 1 in 1000 infants in developed countries. Epidemiological studies have illuminated various demographic factors—such as age, sex, and socioeconomic status—that influence the incidence of SIDS, revealing significant geographic and temporal trends that warrant deeper investigation. Despite extensive awareness campaigns and preventive measures, the underlying causes of SIDS remain elusive, with myocarditis emerging as a potential contributor in many cases. This research paper aims to explore the intricate relationship between myocarditis and SIDS through a multifaceted approach that includes autopsy techniques, external and internal signs observed during postmortem examinations, and histopathological identification of myocarditis markers. By synthesizing the current literature on the frequency of myocarditis in SIDS cases and its association with viral infections, this study seeks to elucidate how effective autopsy methods can distinguish SIDS from other infant mortality causes, while also addressing the limitations of these techniques. Furthermore, it will examine the external signs often present in SIDS cases and the significance of internal organ assessments in elucidating the pathophysiology of SIDS. Lastly, the role of histopathological analysis in identifying key markers of myocarditis will be critically assessed, highlighting the challenges that researchers face in this domain.

The landscape of sudden infant death syndrome (SIDS) has evolved significantly, as evidenced by a genuine and continued decline in SIDS numbers, which can be attributed to shifts in diagnostic practices and increased awareness about safe sleeping environments [25]. Rigorous clinical history reviews, detailed death scene examinations, and comprehensive autopsy testing have contributed to altering the diagnostic profile for infant deaths, ensuring that cases are more accurately classified [25].

In exploring how demographic factors influence the occurrence of sudden infant death syndrome (SIDS), it is crucial to recognize multiple intersecting elements that contribute to its risk. For instance, the occurrence of SIDS is notably higher in families with twins, suggesting that genetic or perinatal factors common in multiple births might elevate the risk [26]. In the Netherlands, male infants show a statistically significant higher risk of SIDS compared to females, highlighting the importance of sex as a contributing demographic factor [26]. Similarly, low socioeconomic status is another crucial demographic element that heightens the risk of SIDS, perhaps due to associated stressors, limited access to healthcare, or environmental factors [26]. In the United States, racial disparities are glaring, with certain ethnic groups experiencing higher SIDS incidence, underscoring the need for targeted interventions in these communities [27]. Furthermore, young maternal age is consistently associated with increased SIDS risk, which may be attributed to inexperience, less access to resources, or other social determinants of health [9,10,11,12,13,14,15,16,17,18,19,20,21,22,23,24,25,26].

### 4.2. Multifactorial Causes of Pediatric Sudden Death: From Autopsy Findings to Prevention Strategies

Sudden cardiac death in children represents a major diagnostic and forensic challenge, encompassing a spectrum of pathological conditions including myocarditis, cardiomyopathies, coronary anomalies, and viral infections. A comparative analysis of 40 reviewed studies underscores the complexity of this phenomenon and the urgent need for accurate, standardized diagnostic protocols. Myocarditis has emerged as a significant yet often underdiagnosed cause of sudden death, with Dettmeyer, deSa, and Smith et al. documenting its frequent occurrence in pediatric cases [47,76,86]. Its silent clinical presentation, as highlighted by Tavora and Okada, complicates premortem diagnosis, while molecular techniques, such as those employed by Shimizu et al., have revealed strong associations between viral infections and myocarditis, deepening the diagnostic relevance [79,87,89]. Similarly, cardiomyopathies and coronary artery anomalies are recognized as leading causes of sudden death in youth, as evidenced by the work of Vassalini, Kon, and Doolan, with Tavora and Ilina stressing that many of these cardiac conditions remain undetected until autopsy [71,74,79,82,84]. Fragkouli et al. further emphasize the prevalence of ischemic heart disease, especially in patients with underlying risk factors [78]. Infections, both viral and bacterial, play a critical role in several cases of sudden death, as reported by Morentin, Dauger, Yagmur, Brady, and Van Reken, reinforcing the importance of incorporating molecular testing into postmortem investigations [72,75,80,103,104]. The use of structured diagnostic approaches and molecular autopsies, promoted by Bakker, Skinner, and Rizzo, has improved detection of underlying causes, while Grasmeyer et al. advocate for immunohistochemistry to enhance the sensitivity of myocarditis diagnosis beyond conventional histology [37,41,70,88]. The topic of sudden infant death syndrome (SIDS) is addressed by Byard and Råsten-Almqvist, who call for clearer diagnostic criteria to distinguish SIDS from other causes [85,92]; Dettmeyer’s findings of enteroviral myocarditis masquerading as SIDS further support this need for advanced investigation [96]. Forensically, Ondruschka et al. highlight the frequent occurrence of CPR-related injuries in pediatric cases, although these are rarely the cause of death, while Byard illustrates that accidental deaths may conceal underlying medical conditions, underscoring the necessity of thorough forensic assessment [73,92]. Rare diseases and autoimmune syndromes, such as incomplete Kawasaki disease described by Pucci and Yajima, or giant cell myocarditis reported by Özdemir-Kara, present additional diagnostic challenges [93]. In conclusion, the integration of molecular and histopathological tools, combined with heightened clinical awareness and standardized postmortem protocols, is essential for elucidating the causes of sudden cardiac death in the pediatric population and for establishing preventive strategies [91,97]. Epidemiological data also indicate a potential increase in the incidence of myocarditis in children during certain viral epidemics. Mounts et al. (2001) described a cluster of fulminant myocarditis cases in pediatric patients during an epidemic in Baltimore, Maryland [106]. These findings support the hypothesis that outbreaks of specific viral agents may contribute to transient surges in myocarditis-related deaths and underscore the need for syndromic surveillance in pediatric sudden deaths

### 4.3. Role of Myocarditis in SIDS

The identification of myocarditis as a contributing factor to sudden infant death syndrome (SIDS) is primarily supported by histopathological and molecular evidence. Research has revealed the presence of viral myocarditis in SIDS cases, as demonstrated by both histopathological examination and polymerase chain reaction (PCR) testing [29]. These investigations have frequently uncovered subtle pathological changes in the cardiac tissues of SIDS infants, suggesting the possibility of underlying viral myocarditis [29]. Additionally, studies have identified nucleic acids from viruses such as enteroviruses, adenoviruses, Epstein–Barr virus (EBV), and parvovirus B19 in the heart tissues of SIDS cases, which are notably absent in control subjects [29]. This viral presence highlights a potential connection between viral infections and myocarditis, contributing to the pathophysiology of SIDS. The integration of this evidence underscores the importance of thorough viral screening in suspected SIDS cases to better understand the role of myocarditis, paving the way for more targeted preventive strategies and potentially reducing the incidence of SIDS through early detection and intervention.

Understanding the frequency of myocarditis in SIDS cases is integral to unraveling the complexities surrounding sudden infant deaths. Sudden infant death syndrome (SIDS) is often a nonconclusive cause of death, with conditions like myocarditis playing a noteworthy role in sudden unexpected death in infancy (SUDI) cases [30,31]. Studies indicate that myocarditis has been identified in only a small proportion of SIDS cases, such as 2% in black SIDS cases and 1% in white SIDS cases [32]. This underscores the necessity for improved diagnostic techniques, such as modern immunohistochemical methods, to better identify myocarditis in SIDS cases and refine our understanding of its prevalence and impact [33,34].

There is ongoing debate regarding the histological criteria required to diagnose myocarditis in the context of infant deaths, reflecting the intricate link between these viral infections and SIDS [35]. Furthermore, findings such as liquid, unclotted blood within the heart chambers in SIDS cases suggest a potential association with myocarditis, pointing to the need for more comprehensive investigations [34]. To unravel these interconnections, an integrated, multidisciplinary approach is crucial, as it can lead to a more nuanced understanding of the etiopathogenesis underlying sudden cardiac deaths related to viral infections and myocarditis [35]. Such an approach may also help address the forensic challenges posed by these conditions, facilitating more effective prevention and intervention strategies.

### 4.4. Autopsy Techniques in SIDS Cases

The exploration of effective autopsy methods in diagnosing SIDS hinges significantly on advancements in both histopathological and immunohistochemical techniques. The anatomo-pathologic examination, which includes the comprehensive study of histological serial sections of critical areas such as the brainstem, cerebellum, and spinal cord, is paramount in identifying any morphological or functional abnormalities that might contribute to SIDS [36]. In addition to examining the central nervous system, the cardiac conduction system is also a focal point, with serial sections taken from the sino-atrial node and the atrio-ventricular system to detect potential anomalies [36]. Furthermore, the integration of immunohistochemical quantification, particularly of specific inflammatory cells, offers a more reliable diagnostic approach for conditions like myocarditis, which has been linked to SIDS [37]. It is evident that adopting a multifaceted approach that combines both traditional and innovative techniques is crucial for increasing the accuracy of SIDS diagnoses and requires continuous refinement of autopsy protocols to keep pace with emerging research and technological advancements [38].

Autopsies play a crucial role in differentiating between SIDS and other causes of infant death by providing a systematic approach to determining the cause of death [39]. The autopsy process involves a comprehensive examination of the infant’s body, which includes both radiological and pathological assessments, though there are challenges in accurately identifying respiratory-related causes due to poor radiological–pathological agreement [39]. Despite these challenges, autopsies can classify causes of death effectively, distinguishing between SIDS, borderline SIDS, and non-SIDS categories [39]. Moreover, autopsies uncover a wide array of pathological findings, with 150 pathological findings identified in a sample of 102 infants, shedding light on various potential causes of death beyond SIDS [39]. These findings are instrumental in guiding pathologists to explore other possible causes such as accidental asphyxia, which is increasingly being diagnosed and differentiated from SIDS through autopsy results [40].

The limitations of current autopsy techniques in SIDS research are multifaceted and significantly hinder the ability to accurately diagnose and understand the underlying causes of sudden infant death syndrome. One major issue is the absence of uniform diagnostic criteria at postmortem, which has resulted in large variations in SIDS rates across Europe and other countries [41]. This lack of standardization complicates cross-country data comparisons and contributes to inconsistencies in reported SIDS incidences. Furthermore, the reliance on histological changes at the alveoli, interstitial tissue, or bronchi to diagnose explained respiratory sudden unexpected infant deaths (SUID) underscores the limitations of these techniques in accurately identifying causative diseases through autopsy [41]. Such reliance may lead to misdiagnosis or incomplete diagnosis, as not all potential causes may be detectable with current methods. Moreover, the current autopsy techniques often overlook major clues provided by pathological findings, which are crucial for gaining insights into SIDS [42]. These overlooked clues suggest that the existing techniques might not be comprehensive enough to capture all relevant physiological changes or anomalies associated with SIDS. To improve the understanding and diagnostics of SIDS, it is essential to enhance and standardize autopsy protocols, ensuring a more thorough examination of pathological clues and potentially revising the classification criteria used in SIDS research.

### 4.5. External and Internal Signs in SIDS Cases

In examining external signs commonly observed in SIDS cases, several pathological findings emerge as significant indicators. One such indicator is the presence of thymomegaly, which is frequently noted in these cases [43]. This enlargement of the thymus suggests a potential imbalance in the immunological development of the infant, which might contribute to the unexplained death. Additionally, SIDS cases often exhibit the presence of liquid unclotted blood in the heart’s chambers, a sign that may reflect a failure in the normal coagulation processes at the time of death [43]. Another critical finding is microcardia, which denotes an abnormally small heart, potentially pointing to underlying developmental issues that could predispose infants to sudden death [43].

This is further supported by the observation that brain weight and head circumference are also greater in SIDS cases, highlighting anatomical anomalies that could be indicative of the syndrome [43]. Moreover, the pattern of petechiae limited to the chest cavity in SIDS cases, as opposed to other causes of sudden death where petechiae may extend below the diaphragm, provides a distinctive marker that aids in distinguishing SIDS from other pathological processes [43]. These organ examination findings suggest a possible prenatal origin of some of these distinctive anatomical features, emphasizing the need for further research into prenatal influences and their role in the development of SIDS [43]. Overall, internal organ examinations offer significant potential in identifying SIDS by revealing specific anatomical and pathological patterns that set it apart from other causes of infant mortality, underscoring the importance of comprehensive postmortem evaluations.

Moreover, genetic studies have become crucial in this context, revealing that familial or genetic diseases could account for up to 35% of SIDS cases, which may not be detectable through traditional autopsy procedures [46]. These insights highlight the multifactorial nature of SIDS, necessitating a combination of genetic and environmental investigations to fully understand the syndrome’s etiology [46]. Therefore, advancements in postmortem genetic testing and functional assessments are essential to enhance the understanding of SIDS and potentially unveil new pathophysiological mechanisms involved in its occurrence [46].

### 4.6. Histopathological Identification in SIDS

Identifying key histopathological markers for myocarditis in cases of SIDS is critical to discerning potential underlying causes. Immunohistochemical methods have emerged as valuable tools in this diagnostic process, allowing the quantification of interstitial lymphocytes and macrophages, which can provide standard values specifically for infants [47]. These methods enable a more precise diagnosis by addressing the limitations of traditional histological approaches, such as the Dallas criteria, which are often plagued by high interobserver variability and sampling error [47]. Although the Dallas criteria, established in 1986, have long represented the standard for histopathological diagnosis of myocarditis in endomyocardial biopsy, their limitations are now well recognized. These criteria define myocarditis as the presence of an inflammatory infiltrate associated with myocyte necrosis not attributable to ischemic injury. However, both sampling error and significant interobserver variability reduce their diagnostic reliability. More importantly, recent research and clinical experience suggest that myocardial necrosis is not an absolute prerequisite for sudden death in infants with myocarditis. Fatal arrhythmias may occur in the context of conduction system involvement even in the absence of histologically evident myocyte damage. As a result, necrosis-free myocarditis—particularly when restricted to functionally critical cardiac areas—must not be overlooked in the differential diagnosis of sudden unexpected infant death. The importance of accurate sampling cannot be overstated, especially in cases with single focal lymphocytic infiltrates, where the number of samples taken during autopsy significantly impacts the diagnostic outcome [47]. Furthermore, molecular pathological techniques, such as the detection of enteroviral capsid protein VP1 and enterovirus RNA through RT-PCR, are increasingly recognized as essential in identifying myocarditis markers [48]. These molecular markers not only support the diagnosis of myocarditis but also highlight the potential role of viral infections in cases previously attributed to SIDS [47]. Taken together, these advanced diagnostic approaches underscore the need for comprehensive molecular and immunohistochemical analyses to improve the accuracy of identifying myocarditis in SIDS, guiding further research and clinical practices to prevent such unexpected deaths.

Histopathological studies play a crucial role in understanding the complex etiology of SIDS by revealing underlying neuropathological and inflammatory processes. Among these, neuropathological features such as neuronal apoptosis and microglial activation provide insights into potential shock-related mechanisms that may contribute to SIDS pathophysiology [49]. Moreover, the presence of low-grade lung and myocardial inflammation, as observed in histopathological examinations, supports the hypothesis that infections could be a significant factor in SIDS, aligning with the infection model proposed by researchers [49]. These inflammatory findings are further supported by the observation of hematogenous shock and shock-like diaphragmatic muscular degeneration, which are typical changes noted in SIDS cases and are indicative of systemic stress or shock [49]. The interplay between these domains underscores the importance of histopathological analysis in identifying potential infectious and immune-mediated mechanisms that may underlie SIDS and highlights the need for continued research to develop targeted preventive strategies.

The histopathological analysis of SIDS cases is fraught with challenges, particularly in distinguishing these cases from other causes of sudden unexplained infant death (SUID) [41]. One significant complication arises from the absence of uniform diagnostic criteria at postmortem, leading to significant variability in reported SIDS rates across different jurisdictions [41]. This lack of consensus complicates the classification of histological findings, which is further exacerbated by the frequent occurrence of acquired airway infections and inflammation in children, muddying the waters between explained and unexplained deaths [41]. Moreover, the reliance on expert consensus documents, particularly in diagnosing conditions such as myocarditis, introduces inconsistencies that can hinder accurate analysis [41].

The findings presented in this study illuminate the multifaceted nature of SIDS and underscore the urgent need for comprehensive public health strategies that address both the epidemiological and biological dimensions of this tragic phenomenon. Furthermore, the exploration of myocarditis as a potential contributing factor to SIDS, supported by histopathological evidence, opens new avenues for research into viral infections and their role in infant mortality. Future research should prioritize the integration of advanced diagnostic techniques and the establishment of uniform criteria, alongside public health initiatives that leverage social media to disseminate evidence-based guidelines effectively. This comprehensive approach could foster a more informed community, ultimately contributing to the reduction in SIDS and improvement in infant health outcomes on a global scale.

### 4.7. Laboratory Techniques for Diagnosing Myocarditis in Infants

One of the most crucial laboratory techniques available for diagnosing myocarditis in infants is the polymerase chain reaction (PCR), which is primarily used to analyze endomyocardial biopsies. This technique enables the precise detection of viral genomes within heart tissue, providing a direct indication of viral infections that may be causing the myocarditis [50]. PCR’s ability to amplify small amounts of DNA to detectable levels makes it a powerful tool in the identification of specific pathogens, thereby aiding clinicians in tailoring appropriate therapeutic interventions. In addition to PCR, reverse transcription PCR (RT-PCR) is employed to investigate the viral etiology of myocarditis, offering insights into the presence of RNA viruses that may not be detectable by standard PCR due to their RNA nature [50]. This dual approach of using both PCR and RT-PCR enhances the diagnostic capability by covering a broader spectrum of potential viral agents, ultimately leading to more accurate diagnoses and better-informed treatment strategies. As myocarditis in infants can often be a life-threatening condition, the integration of these laboratory techniques is vital for early and precise diagnosis, which is essential for improving patient outcomes.

In the context of diagnosing myocarditis related to SIDS, histological examination remains a cornerstone technique, despite some limitations. Histology offers direct visualization of inflammation in the myocardium, which is crucial for confirming myocarditis; however, its effectiveness can be compromised by technical issues and background staining that may obscure results [51]. This can be particularly problematic when attempting to differentiate myocarditis from other potential causes of sudden infant death, as the presence of inflammation does not always correlate with the cause of death [52]. Moreover, while histology can detect myocarditis, it does not always provide a definitive explanation for SIDS, as myocarditis may be present but not necessarily the cause of death [53]. Therefore, while histological examination is an important tool, reliance on it alone without complementary techniques such as PCR may result in incomplete or misleading conclusions regarding the role of myocarditis in SIDS cases. Combining histology with other diagnostic methods could enhance the accuracy and reliability of detecting myocarditis in the context of SIDS, underscoring the need for an integrated approach to diagnosis. Virus-induced lymphocytic myocarditis may also present as a focal myocardial infection. Hence, protocols for postmortem examination must account for this patchy distribution. If sampling is limited, particularly in standard autopsies, there is a substantial risk of underdiagnosing focal myocarditis. We recommend a more extensive and systematic sampling strategy, which includes not only both ventricles and the septum, but also targeted sampling of the conduction system when feasible.

The limitations and challenges associated with diagnostic techniques are multifaceted and pose significant hurdles in accurately identifying diseases like SIDS and sudden intrauterine unexpected death syndrome (SIUDS). One major limitation is the lack of advancement in diagnostic research methodologies, which have not kept pace with the rapid development of diagnostic techniques, leading to methodological flaws and poorly defined research designs [54]. This disparity is also evident in the evaluation of diagnostic techniques, which remains less advanced than the evaluation of treatments, thereby affecting the accuracy and reliability of diagnostic outcomes [54]. Moreover, traditional diagnostic methods are plagued by technical problems such as low sensitivity and specificity in antibody and antigen detection methods, which significantly limits their effectiveness in clinical settings [55]. These technical issues are compounded by the practical challenges faced by amplification-based COVID-19 assays, which encounter both preanalytical and analytical hurdles that can compromise the performance of the tests [55]. Additionally, the financial burden of in-person testing and the risk of overloading healthcare facilities further complicate the implementation of these diagnostic techniques, suggesting the need for more streamlined and cost-effective approaches [55].

### 4.8. Developing an Operational Protocol for Investigating SIDS

To ensure consistency in diagnosing myocarditis as a factor in SIDS, it is imperative to establish a standardized protocol that encompasses both autopsy practices and diagnostic definitions [57]. The introduction of a consistent autopsy protocol, which includes a specific screening test for the detection of myocarditis at autopsy, can improve diagnostic accuracy and reliability [58]. Moreover, the use of standardized scene investigation protocols is vital to complement autopsy findings and provide a comprehensive understanding of each case [59]. These protocols should integrate the current diagnostic standards, such as histological examination [59]. Ultimately, the standardization of diagnostic procedures and criteria is essential to enhance the identification of myocarditis in SIDS cases, facilitating more accurate epidemiological studies and potentially informing preventive strategies.

**Figure 3:**
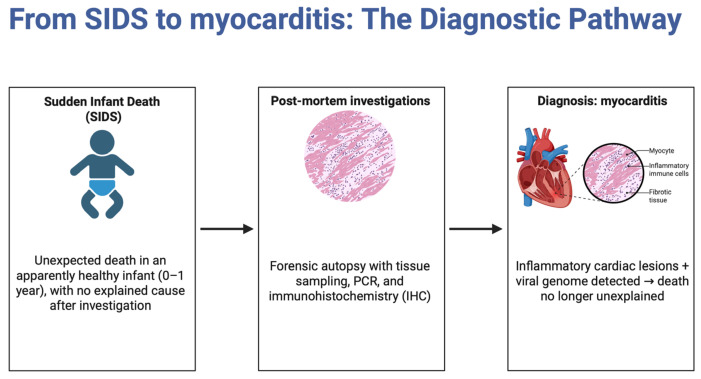
Postmortem diagnostic workflow illustrating how complete autopsy, including histological and molecular analysis, enables the identification of myocarditis as a cause of death in cases initially classified as SIDS (source: Biorender.com).

In investigating cases of SIDS, the identification of myocarditis as a potential underlying cause is crucial, particularly when traditional histological staining methods yield inconclusive results [61]. To enhance the accuracy of diagnosing myocarditis in these instances, modern diagnostic methods must be employed. This includes the utilization of molecular–pathologic investigations such as RT-PCR, which can detect viral genomes associated with myocarditis, thereby strengthening the reliability of the diagnosis [61]. Such protocols must not only increase the number and anatomical diversity of myocardial samples but also ensure the preservation of tissue quality suitable for advanced investigations. In particular, the use of neutral buffered formaldehyde as a fixative and an adequate fixation time of 48 to 72 h are essential to preserve antigenicity and nucleic acid integrity. Suboptimal fixation conditions can significantly impair both immunohistochemical analyses and molecular pathology studies, thus compromising diagnostic yield. Furthermore, immunohistochemical techniques provide additional critical indicators. The use of markers such as Leukocyte Common Antigen (LCA), CD68, CD45R0, and Major Histocompatibility Complex (MHC) class II molecules, along with detection of the VP1 capsid protein of enteroviruses, offers a comprehensive approach to confirm myocarditis presence in SIDS cases [61]. These advanced methodologies not only improve diagnostic precision but also underscore the importance of integrating molecular and immunohistochemical techniques to thoroughly rule out myocarditis, ensuring that SIDS cases are accurately classified and understood [61]. This integrated approach highlights the need for continued advancement and application of sophisticated diagnostic tools in forensic investigations of unexplained childhood deaths (Figure 3).

Emerging technologies, such as advanced imaging techniques and molecular diagnostics, have shown great promise in enhancing the accuracy of ruling out myocarditis in SIDS investigations. One pivotal advancement is the use of novel imaging tracers that can detect early signs of myocarditis, which are often difficult to identify using conventional methods [62]. These imaging techniques, particularly when integrated with comprehensive protocols, allow a more thorough examination of the heart, potentially revealing underlying conditions, such as arrhythmogenic right ventricular cardiomyopathy (ARVC), that may contribute to unexpected infant deaths [63]. Additionally, molecular hybridization techniques have significantly improved the detection of viral infections in cardiac tissues, thus aiding in the identification of myocarditis as a potential contributing factor in SIDS cases [64]. The integration of these technologies into standard SIDS investigation protocols not only enhances diagnostic accuracy but also helps in preventing potential misdiagnoses, thereby reducing the emotional and legal ramifications for affected families [65]. As these technologies continue to evolve, it is essential to incorporate them into routine investigations.

Myocarditis, which can often present without overt symptoms or detectable markers until postmortem analysis, necessitates a meticulous approach to histopathological examinations to ensure accurate diagnosis [66]. The challenge lies in differentiating between myocarditis and other similar pathologies such as hypertrophic cardiomyopathy and inherited arrhythmias, which have been identified in SIDS victims [67]. This differentiation is crucial as it directs the focus towards understanding the broader epidemiology of myocarditis and its role in sudden deaths, including those beyond the infant population [68]. Furthermore, genetic investigations are becoming increasingly significant as they may offer insights into the predispositions that contribute to SIDS, potentially ruling out myocarditis as a direct cause but highlighting its relevance in the context of genetic and metabolic conditions [69]. As we build a more comprehensive understanding of these interconnected domains, it becomes essential for ongoing research to refine diagnostic criteria and improve preventive strategies for SIDS, thereby enhancing the accuracy of forensic investigations and potentially saving lives.

### 4.9. Future Directions

The findings of this review suggest several important directions for future research and clinical practice. First, there is a clear need for the systematic application of standardized autopsy protocols, including histopathology, immunohistochemistry, and molecular diagnostics, in all cases of sudden and unexplained infant death. Such protocols would enhance diagnostic sensitivity for myocarditis and reduce the proportion of deaths classified as unexplained. Second, the identification of myocarditis in postmortem investigations should prompt consideration of familial screening, especially for viral cardiomyopathies or inherited arrhythmogenic conditions such as long QT syndrome. Early cardiological evaluation of siblings and first-degree relatives may offer opportunities for risk stratification and preventive intervention. Third, the integration of forensic findings with clinical databases could improve epidemiological tracking of myocarditis-related mortality and help define pediatric populations at higher risk. Finally, future studies should focus on developing minimally invasive postmortem diagnostic tools—such as targeted molecular panels or blood biomarker assays—to allow broader application of myocardial inflammation screening even in settings where full autopsy is not feasible. These directions underline the potential clinical and public health relevance of myocarditis in pediatric sudden death and argue for closer collaboration between forensic, cardiologic, and infectious disease specialists.

## Figures and Tables

**Table 1:** Original studies (including case reports/case series).

Author(s)	Study Description	Additional Notes	Study Type
Dettmeyer R. [47]	Incidence of lethal lymphocytic myocarditis in neonates and children.	Study highlighting the pathological findings of lymphocytic myocarditis leading to sudden death in pediatric autopsies.	Original research
Vassalini M et al. [71]	Sudden cardiac death in individuals aged 1–40 in Brescia.	Epidemiological study of sudden cardiac death in young individuals; emphasizes need for autopsy and histological analysis.	Original research
Dauger S et al. [72]	Sudden death in an infant with Varicella Zoster infection.	Case of viral myocarditis leading to sudden death; emphasizes the importance of viral screening postmortem.	Original research
Ondruschka B et al. [73]	CPR-related injuries in neonates and children.	Analysis of rib and sternal fractures caused by CPR in infants and their implications in forensic evaluation.	Original research
Kon FC et al. [74]	Cardiovascular deaths in childhood and adolescence.	Comprehensive review of cardiovascular causes of death in young patients, with emphasis on undiagnosed heart conditions.	Original research
Morentin B et al. [75]	Sudden unexpected infectious deaths in children and young adults.	Evaluation of infectious diseases causing sudden death; key findings from autopsy and histopathology.	Original research
deSa DJ. [76]	Three cases of isolated myocarditis in infants.	Three clinical and autopsy cases presenting isolated myocarditis as a cause of death in otherwise healthy infants.	Case report/case series
Diaz FJ, Loewe C, Jackson A. [77]	Analysis of 72 myocarditis cases across all age groups.	A multiage study analyzing clinical and histological findings across 72 myocarditis cases.	Original research
Fragkouli K, Vougiouklakis T. [78]	11 years of autopsies for sudden cardiac death in Greece.	Review of forensic autopsies over 11 years focused on identifying causes of pediatric cardiac arrest.	Original research
Tavora F, Li L, Burke A. [79]	Coronary anomalies as cause of sudden death in children.	Autopsy findings of coronary artery anomalies in children linked to exercise-induced sudden death.	Original research
Yagmur G et al. [80]	Real-time PCR for postmortem diagnosis of infections in infants.	Use of molecular biology tools (RT-PCR) in postmortem detection of pathogens associated with sudden death.	Original research
Grimaldi F et al. [35]	Case of fatal infantile myocarditis.	Report of an infant death due to rapidly progressive myocarditis confirmed on histology.	Original research
Ilina MV et al. [82]	Undiagnosed heart diseases and sudden death in children.	Retrospective review of missed congenital and acquired cardiac diseases in sudden death cases.	Original research
Shatz A, Hiss J, Arensburg B. [83]	Myocarditis misdiagnosed as SIDS.	Postmortem reevaluation showing misdiagnosis of myocarditis as SIDS; implications for cause of death certification.	Original research
Doolan A, Langlois N, Semsarian C. [84]	Sudden cardiac death in young Australians.	National study analyzing causes and genetic risks in youth cardiac arrest; promotes screening in schools.	Original research
Råsten-Almqvist P et al. [85]	Heart weight in SIDS and other causes of death.	Comparison of heart weights in SIDS and non-SIDS cases; questions overdiagnosis based on organ size.	Original research
Smith NM et al. [86]	32 cases of myocarditis in childhood and adolescence.	Series emphasizing myocarditis patterns across age groups, including cellular infiltrates and viral detection.	Original research
Shimizu C et al. [87]	Viruses in SIDS cases with myocarditis and pericarditis.	Demonstrates viral genome presence in myocarditis/pericarditis-related infant deaths.	Original research
Skinner JR et al. [88]	Molecular autopsy for long QT gene.	Genetic autopsy identifying mutations linked to long QT syndrome in cases of sudden unexplained death.	Original research
Okada R, Kawai S, Kasyuya H. [89]	Autopsy cases of nonspecific myocarditis.	Series of autopsy cases without definitive etiology; suggests need for standardized myocarditis criteria.	Original research
Okuni M et al. [90]	Thymus role in chronic pediatric myocarditis.	Histological and clinical correlation of thymic alterations with chronic myocarditis course in children.	Original research
Grasmeyer S, Madea B. [37]	Immunohistochemistry in myocarditis diagnosis from autopsy tissue.	Utility of immunohistochemistry markers (CD3, CD68) for confirming myocarditis in autopsy samples.	Original research
Pucci A et al. [91]	Incomplete Kawasaki disease and sudden death in infants.	Case linking incomplete Kawasaki syndrome to fatal coronary arteritis in infants.	Original research
Byard RW. [92]	Coincidental findings in infant accidental death autopsies.	Highlights accidental findings that can mimic signs of abuse in infant autopsies.	Original research
Özdemir-Kara D et al. [93]	Case of giant cell myocarditis in a neonate.	Unique case of giant cell myocarditis in neonate; underscores importance of early diagnosis and biopsy.	Original research
Savage TR, Smith JF. [94]	Polyarteritis nodosa and congenital pyloric hypertrophy in an infant.	Combined occurrence of autoimmune vasculitis and gastrointestinal anomaly in a fatal infant case.	Original research
Dettmeyer R et al. [95]	Enteroviral myocarditis diagnosis in SIDS.	Evidence of enteroviral infection in SIDS case with myocardial inflammation confirmed by PCR.	Original research
Jedidi M et al. [96]	Acute myocarditis mimicking myocardial infarction in a neonate.	Neonatal myocarditis clinically mimicking myocardial infarction; confirmed via autopsy.	Original research
Yajima D et al. [97]	Sudden death in an infant due to incomplete Kawasaki disease.	Infant death due to vasculitis-like Kawasaki pathology with cardiac involvement.	Original research
Puffer P. [98]	Sudden cardiac death in childhood and adolescence.	Autopsy-based review showing arrhythmogenic mechanisms in pediatric sudden death.	Original research
Weber MA et al. [99]	Pediatric myocarditis in autopsy series.	Survey of myocarditis prevalence and features in forensic autopsies of children.	Original research
Grant EK, Evans MJ. [100]	Cardiac findings in fetal and pediatric autopsies.	Findings from congenital and acquired cardiac malformations in perinatal and pediatric autopsies.	Original research
Partoune B et al. [101]	Analysis of causes of sudden death in children.	Broad overview of sudden death causes in children across a multicenter autopsy dataset.	Original research
Dettmeyer R et al. [102]	Myocarditis in suspected SIDS cases.	Postmortem myocardial analysis in suspected SIDS cases reveals inflammatory changes.	Original research
Brady MT et al. [103]	Neonatal death with AIDS and CMV pancarditis.	AIDS-related neonatal death associated with CMV pancarditis; confirmed immunohistochemically.	Original research
Van Reken DE et al. [104]	Congestive heart failure in infant with echovirus infection.	Case of fatal viral myocarditis due to echovirus with signs of cardiac failure in infant.	Original research
Lajoie J et al. [105]	Reye’s syndrome with acute myocarditis in an infant.	Report of Reye’s syndrome associated with myocardial involvement in autopsied infant.	Original research

**Table 2:** Review and methodological studies.

Author(s)	Study Description	Additional Notes	Study Type
Bakker AM et al. [70]	Diagnostic protocol for sudden cardiac arrest in children.	Proposed standardized diagnostic workflow for evaluating sudden cardiac arrest in pediatric emergency settings.	Review/methodological
Byard RW. [81]	Definitions of sudden infant death syndrome (SIDS).	Discussion of evolving definitions and diagnostic challenges in SIDS classification and reporting.	Review/methodological
Rizzo S et al. [41]	Postmortem protocol for sudden infant death syndrome (SIDS).	Proposal of a thorough postmortem protocol integrating molecular and histological diagnostics in SIDS.	Review/methodological

## Data Availability

Not applicable to this article as no datasets were generated.
